# Nutraceutical and Medicinal Potential of the *Morus* Species in Metabolic Dysfunctions

**DOI:** 10.3390/ijms20020301

**Published:** 2019-01-14

**Authors:** Elisana Lima Rodrigues, Gabriela Marcelino, Gabriela Torres Silva, Priscila Silva Figueiredo, Walmir Silva Garcez, Joaquim Corsino, Rita de Cássia Avellaneda Guimarães, Karine de Cássia Freitas

**Affiliations:** 1 Post Graduate Program in Health and Development in the Central-West Region of Brazil, Federal University of Mato Grosso do Sul-UFMS, Campo Grande, MS 79079-900, Brazil; elisana.lima10@gmail.com (E.L.R.); gabi19ac@gmail.com (G.M.); gabitorres483@gmail.com (G.T.S.); pri.figueiredo92@gmail.com (P.S.F.); ritaaguimaraes@gmail.com (R.d.C.A.G.); 2 Chemistry Institute, Federal University of Mato Grosso do Sul-UFMS, Campo Grande, MS 79079-900, Brazil; walmir.garcez@ufms.br (W.S.G.); corsinojoaquim@gmail.com (J.C.)

**Keywords:** Moraceae, medicinal plants, antioxidants, flavonoids, diabetes mellitus

## Abstract

Many populations use medicinal plants as a therapeutic treatment, due to their lower cost and greater access. Among the plant species used for medicinal purposes are those of the genus *Morus*. The most known species are *Morus alba*, *rubra*, and *nigra.* This review aims to collect data from the literature, predominantly from cell and animal studies, which presents a possible nutraceutical and medicinal potential of the species *Morus* for use in metabolic dysfunctions. The fruits and leaves of mulberry are used for therapeutic purposes. For scientific confirmation of these effects, they were studied for laxative properties, antibacterial activity, anti-atherogenic activity, and hepatoprotective function. Furthermore, the genus *Morus* is recognized for the treatment and prevention of diabetes mellitus, through its hypoglycemic action. It may also provide health benefits through immunomodulatory, anti-inflammatory, and anti-nociceptive effects. It has been found that the *Morus* species have phenolic compounds, flavonoids, and anthocyanins that act as important antioxidants and promote beneficial effects on human health. These phytochemical compounds differ among species. Blackberry (*Morus nigra*) are rich in flavonoids, while the white mulberry (*Morus alba*) has low concentrations of flavonoids and anthocyanins. In addition, another important factor is to ensure a complete exemption of toxic risks in the use of medicinal plants for the treatment of diseases. Studies have shown no toxic effects by the administration of extracts of *Morus* species. Thus, the mulberry tree presents nutraceutical potential. It is therefore a promising alternative for medicinal products based on medicinal plants.

## 1. Introduction

Medicinal plants have been used as a therapeutic treatment since 4000–5000 B.C., with reports in different populations, mainly Asian, and applications in the treatment and prevention of different disorders, such as inflammatory processes, intestinal diseases, cutaneous conditions, and even cancer [1].

The safety and efficacy of medicinal plants have been well-accepted, as seen in traditional long-term use and scientific research [2]. Because of their accessibility, especially compared to modern drugs, plants have become an important part of the primary healthcare system [2].

Due to the wide variety of species and effects related to their use, studies have sought to identify the mechanisms involved in these processes, mainly those that are related to chronic non-communicable diseases, such as obesity and diabetes, since many plants have antidiabetic effects [1,3], among other effects that act in the fight against such diseases.

Among the species of plants used for medicinal purposes are those of the genus *Morus*, popularly known as mulberry, which belongs to the family Moraceae. Three species are best known: *Morus alba*, *rubra*, and *nigra*. This species is a mono- or dioic plantar of small- to medium-size, widely distributed in India, China, Japan, North Africa, Arabia, and southern Europe, among other regions [4].

The species *Morus* is a rich source of phenolic compounds, including flavonoids and anthocyanins, of great biological, pharmacological, and structural interest because of their antioxidant properties [4]. Traditionally, the species are used for the prevention of liver and kidney diseases, joint damage, and anti-aging, due to their antioxidant properties [5]. In addition, it has been shown to be an ally in the treatment of type 2 diabetes mellitus (DM2), due to its hypoglycemic effects [6].

Both obesity and DM2 are characterized by high serum free fatty acid (FFA) concentrations, reflecting an increased infiltration of macrophages in white adipose tissue (WAT) and lower insulin sensitivity [7]. In addition to increased FFA levels, other active metabolites are involved in obesity, such as ceramides, diacylglycerols, and acetyl-coA, which act by stimulating protein kinases, such as N-terminal c-Jun kinase (JNK), protein C-kinase (PKC), and nuclear factor-κ B (NFκB) inhibitor, which are responsible for impairing insulin sensitivity by increasing inhibitory phosphorylation [8,9].

Insulin resistance (IR) is one of the major triggers of DM2 and is considered an important co-morbidity that is related to obesity and metabolic syndromes. The IR process involves a reduction in glucose uptake to peripheral tissues, overproduction of glucose by the liver, functional damage to pancreatic β-cells, and a decrease in the mass of β-cells [10].

Leaves of various varieties of the species *Morus* have a high concentration of sugar-mimicking alkaloids known to have hypoglycemic properties, such as 1,4-dideoxy-1,4-imino-D-arabinitol, 1-deoxynojirimycin, and 1,4-dideoxy-1,4-imino-D-ribitol [11]. They are able to inhibit all or some intestinal disaccharidases and pancreatic amylases by regulating the uptake of monosaccharides, and are therefore therapeutically used in the oral treatment of type 2 diabetes mellitus [12]. 

In addition, mulberry leaf extract is able to attenuate RI by modulating gene and protein expression involved in glucose homeostasis in liver cells. The activities of the gluconeogenic enzymes phosphoenolpyruvate carboxykinase (PEPCK) and glucose-6-phosphatase (G-6-Pass) are suppressed, whereas the activities of glycolytic enzymes (glucokinase (GK), phosphofructokinase (PFK), and pyruvate kinase (PK)) are stimulated, depending on the dose. In addition, the phosphatidylinositol-3-kinase (PI3K)/protein kinase B (AKT) and glycogen synthase kinase-3β (GSK-3β) signaling pathways are activated by elevating the translocation of the glucose transporter (GLUT-4) in skeletal muscles and in adipose tissue [13].

Medicinal hypoglycemic agents, such as metformin and rosiglitazone, are used to regulate protein kinases activated by adenosine monophosphate (kappa), which is responsible for energy control in cells, playing a central role in regulating glucose uptake and insulin sensitivity [14]. However, such medications may cause side-effects, such as liver or cardiovascular problems. Thus, there is a growing search for innovative therapies that are derived from natural compounds with hypoglycemic action as alternatives, or as adjuncts to conventional drug treatment [15].

Considering the appeal for natural compounds as alternatives to therapeutic treatments in obesity-related metabolic disorders, especially DM2, it is important to evaluate their biochemical and molecular pathways of action, as well as to know their long-term effects. Thus, the objective of this review is to discuss the potential of the genus *Morus* as a nutraceutical or medicament for metabolic dysfunctions, emphasizing its importance for the treatment of DM2, evaluating its chemical composition, nutritional properties, toxicity, and mechanisms of action. 

## 2. The Species *Morus*: Nutritional Properties

### 2.1. Chemical Composition

**Figure 1:**
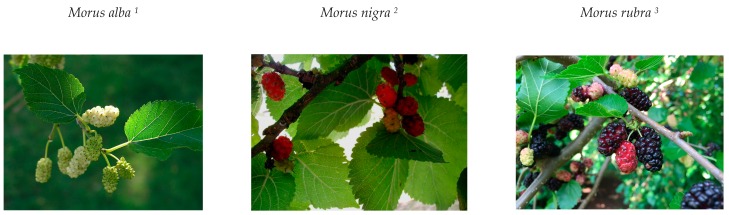
The most common species of the genus *Morus*. Source: ^1^ https://commons.wikimedia.org/wiki/File:Morus-alba.jpg; ^2^ https://commons.wikimedia.org/wiki/File:Morus-nigra.JPG; ^3^ https://commons.wikimedia.org/wiki/File:2017-05-29_14_12_27_Red_Mulberry_fruit_along_Kinross_Circle_in_the_Chantilly_Highland_section_of_Oak_Hill,_Fairfax_County,_Virginia.jpg.

There are 24 *Morus* species, and at least 100 varieties of known subspecies that adapt to varying climatic, topographical, and soil conditions. Such environmental conditions also influence their compositions, which stand out due to the presence of compounds considered as nutraceuticals, since they provide health benefits and act in the treatment of diseases [16]. The most common species are *Morus alba* (white mulberry), *Morus nigra* (blackberry), and *Morus rubra* (red berry), (Figure 1) [16,17].

In relation to the physical–chemical composition, the species *M. alba*, *M. nigra*, and *M. rubra* present fruits with an average weight between 2.0 and 4.0 g, and a high water content (about 70%). Among the three species, *M. alba* presents the highest values of pH and soluble solids, indicating a sweeter taste, and it is therefore the most recommended for processing. As for acidity values, there was a similarity between *nigra* (1.40%) and *rubra* (1.37%), differing only from white (0.25%) [16].

The lipid content is low in all species; *M. alba* has the highest lipid values (1.1%) [16,18]. The lipid values were higher in mulberry leaves than in fruits, and *M. alba* had the highest percentage of lipids (6.57%), followed by *M. nigra* and *M. rubra* (5.13% and 4.24%, respectively) [19].

With regard to lipid quality, the fatty acid (FA) profile is mostly linoleic (C18:2) for all species, followed by palmitic acid (C16:0), and a lower content of oleic acid (C18:1), found only in *M. nigra* and *M. alba* [16,18].

In relation to the protein content, the fruits of the species *M. nigra* present values of between 8.9 and 10.85%, whereas the species *M. alba* has higher values (between 10.15 and 13.33%). This shows that the species *Morus* represents a good source of plant protein that is capable of contributing to the daily recommended intake of proteins [20].

There is a prevalence of Ca, Mg, Fe, Na, Mn, Zn, Cu, and Se [18] by the evaluation of the minerals that are present in the fruits. In addition, the minerals N, K, and P are found in large quantities [16]. Among the minerals in leaves, *M. alba* and *M. nigra* have high iron values (119.3–241.8 mg/kg) and low sodium values (0.01 mg/100 g), and are a good option for individuals who have sodium restrictions in their diet. Other minerals are also found at lower concentrations, such as zinc, calcium, potassium, and magnesium [6].

In relation to antioxidant properties, the presence of ascorbic acid (vitamin C) stands out, which, among its main functions, prevents and reduces oxidative damage in the organism. Blackberry presents 48.4 mg/100 g of ascorbic acid, approximately eight times higher than white mulberry [18]. In contrast, Ercisli and Orhan [16] reported significantly lower values: 22.4 mg/100 mL for white mulberry, 21.8 mg/100 mL for blackberry, and 19.4 mg/100 mL for red berry. Table 1 presents the main nutrients found in the fruits of *Morus nigra*, *alba* and *rubra*.

### 2.2. Phytochemicals

Phytochemicals act as important antioxidants, presenting beneficial effects on human health, especially in the prevention of cardiovascular, inflammatory, and cancer diseases. Among the phytochemical compounds found in the genus *Morus*, there are mainly flavonoids. Flavonols are groups of flavonoids that are present in the species *M. alba* and *M. nigra*. They are divided into 20 types, whose main glycolyzed forms are quercetin, kaempferol, and isoramnetin, which aid in physiological processes in plants, and they act by reducing the risk of DM2 and some types of cancer [5]. The presence of phytochemical compounds is very evident. The species *M. nigra* and *M. rubra* have 1.422 and 1.035 mg gallic acid equivalents (GAE)/100 g, respectively, for total phenols, and 276 and 219 mg quercetin equivalents (QE)/100 g, respectively, for flavonoids [16]. Table 2 presents the main studies and phytochemicals found in fruits of *Morus nigra*, *alba* and *rubra*.

The species *Morus nigra* originate in the Far East region and is widely used in traditional Chinese medicine as a hepatoprotective, hypotensive, antipyretic, analgesic, diuretic, expectorant, and anti-diabetic [21]. A substantial number of phenolic compounds, which may be responsible for antibacterial and antioxidant activities, was observed in a study on the leaves of *Morus nigra* [22].

Regarding the chemical composition and pharmacological properties of *Morus nigra*, its leaves have a high content of flavonoids, tannins, coumarins, polyphenols, and triterpene and steroid substances, which are capable of exerting an estrogenic or progesteronic effect [23,24].

The species *Morus alba*, popularly known as white mulberry, belongs to the family Moraceae. It is found in temperate and subtropical regions of Asia, the Americas, Europe, Africa, and India. Due to the wide geographic distribution, this species presents a great degree of environmental variability that interferes with its physical and chemical characteristics, mainly with the profiles of bioactive compounds such as anthocyanins, carotenoids, and flavonoids [16,25]. It is a deciduous plant, reaching 10–20 m in height, which is used for landscaping and gardening in urban areas, and even for the stabilization of sandstones [26]. In most Asian countries, it is cultivated for its foliage, which is used as food for silkworms, as well as for the feeding of herbivorous animals, due to its high nutritional value (mainly proteins), as well as having a pleasant taste [6].

The red berry (*Morus rubra* L.), is a deciduous tree of small to medium size, 15–20 m in height, and with leaves measuring between 8 and 15 cm with serrated edges. Its fruits are composed of several drupes, with a dark reddish color and a sweet taste [27]. Like the other species of *Morus*, it also has medicinal properties [28]. Its aqueous extract is not considered toxic, and it exerts effects that are similar to glibenclamide, an anti-hyperglycemic, significantly reducing the level of blood glucose. It is also able to act as a protector in lipid peroxidation, causing a decrease in serum and hepatic malonaldehyde (MDA) levels, an increase in the activity of antioxidant enzymes, and a reduction in glutathione (GSH) [29].

The values of phenolic compounds and flavonoids differ among species due to their genetic characteristics, environmental conditions, and stages of maturation [16]. Blackberry are rich in these antioxidant compounds, whereas white mulberry has low values of flavonoids and anthocyanin values [30]. Cyanidin-3-*O*-glucoside and cyanidin-3-*O*-rutinoside are the main anthocyanins found in these species, since rutin is the main flavonoid [31].

Among the species *Morus alba* and *Morus nigra*, there are derivatives of benzoic acid (protocatechuic acid, *p*-hydroxybenzoic acid, and vanillic acid) and of cinnamic acid (chlorogenic acid and neo-chlorogenic acid) [20]. Other compounds identified in smaller fractions were the derivatives of gallic acid, *p*-hydroxibenzoic acid, caffeic acid, *p*-coumaric acid, and ellagic acid [26].

The leaves also have important nutritional properties that allow for their use in several types of preparations. In the species *Morus alba* and *Morus nigra*, the main organic acids that are found are citric and malic acid, the first one at the highest concentrations (32.2–105.5 mg/100 g). In addition, the leaves of *Morus alba* and *Morus nigra* have a high content of proteins (13.4–19.4%), which allows for their use in wheat flour to increase the nutritional value of the food, besides improving stability during storage [6]. 

The phenolic extract of Morus alba leaves showed antioxidant activity in vitro, which shows a positive correlation with its total phenolic content of 5.55 mg GAE/g dry weight (DW) and the total flavonoid content of 16.96 mg rutin equivalents (RE)/g DW. The main compounds identified that contributed to the antioxidant capacity were chlorogenic acid, rutin, and catechin [32].

In relation to the methanolic and acetonic extracts obtained from the fruits of *Morus nigra*, both showed a higher total antioxidant activity than the fruit extracts of *Morus alba*. As for the total phenolic compounds, the highest value was found for the acetone extract of *Morus nigra* (173 mg/g), followed by the methanolic extract of this same species (164 mg/g); the lowest value was obtained for the methanolic extract of *Morus alba* (119 mg/g) [33].

In relation to the antioxidant power of the ethanolic extract of different parts of the *Morus* species, the leaves of *Morus nigra* had the highest content, followed by the roots of *Morus alba* and *nigra*. The extracts of the fruits had the lowest values [34]. Despite the antioxidant potential of these species, it is important to consider whether there is toxicity in their parts, and to evaluate the safety of their use for inclusion in the diet.

## 3. Toxicity of the Species *Morus*

The validity of the use of medicinal plants for the treatment of diseases has been studied, due to their lower cost and greater access by the population. However, their actions do not ensure a complete exemption of toxic risks, with hepatotoxicity being one of the most common problems. Thus, it is important that studies observe the doses and their side effects to ensure the best benefits without risks to those who consume them [35].

The crude ethanolic extract from the leaves of *Morus nigra* did not show an expressive anti-proliferative effect in the evaluation of cytotoxicity; that is, it demonstrated an absence of cytotoxic activity [22]. Similarly, the intragastric administration of *Morus alba* ethanolic extract in rats at the maximum dose of 1000 mg/kg did not cause changes in the behavior of the animals, such as respiratory changes, weight loss, or death, within one week of continuous administration, indicating that there was no acute toxicity in this type of extract and dose [25].

Administration of higher doses has also been reported by other studies. The effects of the ethanolic extracts from the leaves of *Morus alba* at five different doses (125, 250, 500, 1000, and 2000 mg/kg) in male and female rats, four times over 14 days of administration (days 0, 10, and 14), and did not cause behavioral changes in the animals, nor were there deaths at any of the doses given [36].

The toxicity of the ethanolic extract from leaves of *M. alba* given intra-peritoneally in two doses (300 and 2000 mg/kg) was evaluated for 14 days in Swiss rats. The authors did not observe behavioral changes in the animals, nor were there any deaths. However, changes in the hematological analysis were observed. Both extracts reduced the hematocrit, hemoglobin, mean corpuscular volume (MCV), mean corpuscular hemoglobin (MCH), mean corpuscular hemoglobin concentration (MCHC), lymphocytes, and monocyte rates, and increased the proportion of segmented leukocytes, indicating their influence on the immune system [35]. In another study, the maximum dose of 2000 mg/kg, when given orally, resulted in hematological changes, reducing MCV and MCHC in addition to causing liver changes. On the other hand, the 300 mg/kg dose was shown to be safer without presenting such results [37].

In genotoxicity assays, doses below 300 mg/kg given orally did not result in differences in the number of micro-nucleated polychromatic erythrocytes when compared to the control group. In addition, there was an increase in micronuclei in groups that received the extract, but this did not differ from the negative control, indicating that the ethanolic extract from the leaves of *Morus alba* given orally can be considered safe [35].

These results suggest that the route of administration and the dose of *Morus* leaf extracts directly interfere with the levels of toxicity offered by the plant, and that oral administration is the most highly recommended. This emphasizes that the extracts can be included in the diet safely. Thus, the therapeutic effects evaluated to date, especially at the cellular level, and within in vivo studies, are approached below, considering different metabolic applications.

## 4. Therapeutic Use of the Species *Morus*

The medicinal properties of the *Morus* species are popularly recognized in several countries [21,38]. Parts of the mulberry tree, such as fruits and leaves, were studied to test their therapeutic purposes.

The methanolic extract produced from the dried fruit of *Morus nigra* at two different concentrations, 30 and 70 mg/kg, was used to test for laxative properties in BALB/c mice of both sexes. The functionality of the extract was evidenced by prokinetic and laxative activities, possibly mediated by the stimulation of muscarinic receptors through a combination of the Ca^2+^ channel blocks and anticholinergic effects [39].

Scientific evidence further suggests that the anthocyanins and flavonoids present in the fruits of *Morus nigra* have anti-nociceptive and antibacterial activities against some microorganisms, including *Escherichia coli*, *Pseudomonas aeruginosa*, and *Staphylococcus aureus*. These activities could be related to the inhibitory effects on pro-inflammatory cytokines, inducible nitric oxide synthase (iNOS), and NF-kB [30].

The anti-proliferative capacity and the pro-apoptotic effect of the dimethyl sulfoxide extract of *Morus nigra* (DEM) was investigated, pointing to the development of new natural therapeutic products against adenocarcinoma cells, since it acts by blocking the cell cycle of PC-3 by inducing the activity of caspases 3 and 7 during the G0/G1 phases. This significantly reduces the number of cells at the S phase, which is important because the cells will not reach the following stages, which involve protein and DNA synthesis [40]. 

Obesity is one of the greatest public health problems in the world and treatment options remain very limited due to the side effects associated with conventional therapies. In this sense, the beneficial effect of a standardized composition (UP603) composed of extracts of *Morus alba*, *Ilex paraguariensis*, and *Rosmarinus officinalis* was evaluated in diet-induced obese C57BL/6J mice. It was observed that the animals treated with UP603 showed a decrease in body weight gain, reductions of 65.5% and 16.4% in insulin and leptin, respectively, and a 2.1-fold increase in ghrelin level. In addition, there were reductions of 7.9–21.1% in total cholesterol, 25.4–44.6% in triglyceride, and 22.5–38.2% in low-density lipoprotein (LDL) cholesterol in mice treated with 450–850 mg/ kg UP603 [41].

**Figure 2:**
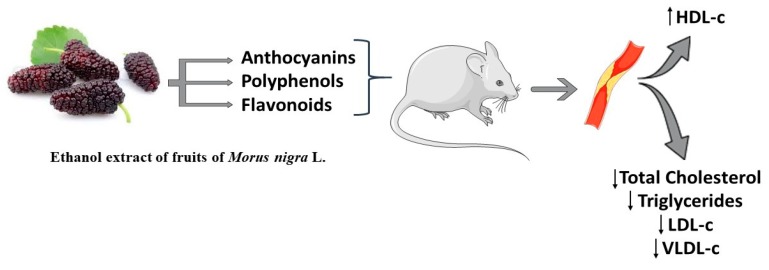
Anti-atherogenic activity of the ethanolic extract from fruits of *Morus nigra* L.

The serum levels of total cholesterol, triglycerides, low-density lipoprotein-cholesterol (LDL-c), and very-low-density lipoprotein-cholesterol (VLDL-c) decreased by the use of *Morus nigra* (210 mg/kg of body weight) fruit ethanolic extract. There was also an increase in the levels of high-density lipoprotein cholesterol (HDL-C) and anti-oxidative enzymatic activities, and a reduction of arterial atherosclerotic lesions in Sprague–Dawley rats fed with a hyper-lipid diet, making it an important option for the prevention and treatment of atherosclerosis [42]. This factor is attributed to its nutritional properties, such as the presence of anthocyanins or the joint action of anthocyanins, polyphenols, and flavonoids (Figure 2) [42,43].

The antioxidant activity present in *Morus* may influence lipid oxidation. The ethanolic extract of blackberry fruits eliminates free radicals, including the 2,2-diphenyl-1-picrylhydrazyl radical, hydroxyl, and superoxide anions, and it has a moderate ability to inhibit the oxidation of linoleic acid [44]. In addition, flavonoids, specifically rutin, have positive effects on lipid peroxidation, being able to decrease the content of thiobarbituric acid-reactive substances (TBARS), and to increase the activity of superoxide dismutase (SOD) and the enzyme glutathione peroxidase (GSH-Px) [21]. 

As it is a serious public health problem, drug-induced liver injury has become a challenge for health professionals, the pharmaceutical industry, and drug regulatory agencies [45]. The flavonoids quercetin, luteolin, and isorhamnetin, present in the leaves of *Morus nigra*, exert hepatoprotective effects. This is because the hydromethanolic extract that evaporated at 70 degrees from the leaves of this mulberry tree produced a significant reduction in the liver enzymes alanine transaminase (ALT), aspartate transaminase (AST), alkaline phosphatase (ALP), and total bilirubin in Swiss mice with paracetamol-induced hepatotoxicity [46].

Non-alcoholic fatty liver disease is characterized by excessive accumulation of lipids in hepatocytes, thus it is one of the most common complications of obesity. The ethanolic extract of *Morus nigra* fruits improved the hepatic steatosis induced by a hyperlipid diet in C57BL/6J mice by a significant reduction in the presence of lipid droplets in hepatocytes, and a decrease in serum levels of ALT and AST and liver levels of triglycerides and total cholesterol. In addition, the protective effects of the extract were associated with improved glucose tolerance, insulin resistance, and insulin sensitivity, as well as induction of fatty acid oxidation and decreased fatty acid and cholesterol biosynthesis [47]. 

In addition, due to the antioxidant and cytoprotective characteristics of leaves of *Morus nigra*, a study reported the inhibition of the growth of cancerous cells in vitro and in experimental animals treated with methotrexate, in combination with the hydroethanolic extract of mulberry leaves. There was a significant decrease in the activities of ALT, AST, and lactate dehydrogenase (LDH), indicating protection against possible hepatic injuries induced by methotrexate, a drug that accumulates in the liver, where it is metabolized and stored as polyglutamate [48].

Popularly, fruits, roots, and leaves of *Morus alba* are used for the treatment of dizziness, insomnia, premature aging, and DM2. They also have a protective effect against atherosclerosis, liver and kidney disorders, and inflammation [25]. The anti-inflammatory activity of the flavonoids present in the mulberry tree, especially quercetin-3-*O*-glycoside, is attributed to the regulation of the expression of genes that are related to inflammation, such as iNOS and COX_2_, by deactivating NF_k_B [49].

Mori Cortex Radicis (MCR) extract dose-dependently reduced serum levels of total cholesterol, triglycerides, and LDL-C, as well as inhibited the activity of ALT, AST, and increased HDL-C in hyperlipidemic Wistar rats. In addition, in vitro biochemistry tests revealed that four active isolates had moderate inhibitory activity against DGAT1, a key enzyme closely related to hyperlipidemia and type 2 diabetes. These results demonstrated that MCR extract can provide a new pharmacological basis for the treatment of hyperlipidemia and diseases related [50].

People in Asian countries, such as Japan and Korea, use the leaves of *Morus alba* and *Morus nigra* as an infusion due to their medicinal properties, which aid in the treatment of diabetes or liver diseases [6]. The use of extracts from other parts of the plant, such as the branches, is beneficial for the treatment of diseases, especially diabetes, due to their hypoglycemic effects [15].

## 5. Therapeutic Use of the Species *Morus* for DM2

DM is a chronic metabolic disorder characterized by hyperglycemia and changes in the metabolism of carbohydrates, proteins, and lipids. It is associated with increased oxidative stress and damage to the pancreatic beta cells, which affect insulin production and the maintenance of stable levels of glucose in the body [51]. This insulin sensitivity is related to the activation of the protein kinase by adenosine monophosphate (AMPK), which regulates glucose uptake and the energetic homeostasis of the body. In addition, its activation increases the expression of the glucose transporter protein 4 (GLUT4), which reduces hepatic gluconeogenesis and improves insulin sensitivity [14]. 

For the treatment of type 2 DM, the use of hypoglycemic agents, such as metformin and rosiglitazone, is necessary to regulate AMPK, and thus decrease blood glucose and improve parameters that are related to hyperlipidemia. However, the use of these drugs may cause side-effects, such as hypoglycemia and poor control of postprandial glycaemia, among others, and thus, there is interest in the search for natural options that minimize such damages [16]. 

After 10 weeks of administration to Streptozotocin-diabetic induced mice, the mulberry leaf powder (*Morus alba*) had a positive effect on fasting blood glucose and insulin levels. The results of Western blot validation exhibited dynamic changes in proteins, such as IGF2, Ly6a, Grb10, and UBD, which may indicate alteration of the insulin receptor substrate (IRS) signaling pathway [52].

Several studies were carried out with the *Morus* species in order to test their effects on the treatment of DM2, especially by using the species *Morus alba* L., as shown in Table 3.

The *Morus* species are recognized for the treatment and prevention of DM2 by its hypoglycemic action, as it increases AMPK and GLUT 4 levels in the plasma membrane [14]. Other mechanisms may still be involved in this process, as well as specific compounds, such as 1-deoxynojirimycin (DNJ), which inhibits α-glucosidase, which, in turn, decreases carbohydrate uptake and thus hyperglycemia [56].

A reduction of fasting and postprandial glycaemia was observed in the treatment with ethanolic extract of *Morus nigra* (400 mg/kg/day), which also improved oral glucose tolerance and reduced lipolysis and proteolysis in diabetic rats induced by aloxane. In addition, the extract reduced the marker for oxidative stress by malondialdehyde, and increased levels of the antioxidant glutathione in the liver of animals. The authors relate the results, possibly, to the identified flavonoids rutin, isoquercetin, and kaempferitrin [43].

Treatment with a hydroethanolic extract of *Morus nigra* L. leaves improved oxidative stress in alloxan-induced diabetic rats, promoting a reduction in the expression and activity of the metalloproteinase matrix (MMP-2), and the superoxide dismutase/catalase ratio. In addition, the extract reduced blood glucose and increased insulin levels. These effects are due to the antioxidant activities of polyphenol compounds that are present in mulberry [58].

Sharma et al. [29] investigated the antidiabetic potential of *Morus rubra* L. leaves, where an antidiabetic action of the aqueous extract of red berry leaves was reported, due to its effect on hyperglycemia, dyslipidemia, and oxidative stress in diabetic Wistar rats induced by streptozotocin. The extract at 400 mg/kg produced a remarkable glycemic control evidenced by the significant reduction in hemoglobin glycosylation with the increase in plasma insulin and C-peptide levels. In addition, it led to a reduction in fasting blood glucose. Furthermore, changed serum lipids in diabetic rats were significantly normalized. There was a reduction in triglycerides, total cholesterol, and LDL-c levels, and an increase in HDL-c levels after treatment with the aqueous extract of *Morus rubra* L., resulting in the control of dyslipidemia.

In addition, according to the aforementioned results, the antioxidant nature of the extract was observed in the erythrocytes and the livers of animals. There was a significant increase in the activity of superoxide dismutase and catalase enzymes, as well as a reduction in lipid peroxides and glutathione peroxides [29]. The normalization of glycaemia in diabetic subjects is fundamental for treatment, since frequent exposure of tissues to high glucose levels may lead to intracellular changes [56].

The effects of ethanolic extracts of polysaccharides extracted from fruits of *Morus alba* were observed in diabetic rats for seven weeks. The animals were divided into four groups: the control group, treated with pure water, treatment with metformin hydrochloride solution (250 mg/kg), and two treatment groups receiving 400 mg/kg of extract with 50% of polysaccharide and 90% of polysaccharide, respectively. As a result, the animals treated with both extracts showed a reduction of 31.9 and 47.5% of the fasting glucose levels, when compared to the control diabetes group. Both extracts affected the lipid metabolism of the animals, leading to a reduction in total cholesterol and triglycerides, an increase in HDL cholesterol, and improved liver function by reducing serum alanine transaminase [25].

Choi et al. [14] also observed a reduced fasting blood glucose level and an increased insulin sensitivity in diabetic rats receiving methanolic extract (15 mg/kg/day, 0.5%, *w*/*w*) of fruits of *Morus alba* over six weeks. These effects were higher than those found for the group receiving hypoglycemic rosiglitazone (RG), which was associated with an increase in AMPK and GLUT4 levels, and resulted in a greater uptake of glucose by the skeletal muscles, and thus, an improvement in hyperglycemia. These results were attributed to the presence of phytochemical compounds, mainly anthocyanins, which have the potential to decrease blood glycaemia and inhibit the development of complications that are associated with diabetes.

The ethanolic extract of *Morus alba* branches, and its main bioactive compound, oxyresveratrol, were studied as for their hypoglycemic effects. The reduction in fasting and plasma glucose levels were attributed to the increase in the glucose transporter type 2 (GLUT-2), and the stimulation of hepatic glucose uptake and glycogen storage. These effects were promoted by oxyresveratrol [15].

White mulberry leaves have nutritional properties. There is a high content of polysaccharides, which increases insulin sensitivity and protects the pancreatic islets from the effects of DM, improving glucose metabolism and protecting the body against damage by oxidative stress. In addition, they improve lipid metabolism, since their deregulation results in changes in insulin levels and glucose metabolism [25,57].

In a study using diabetic rats fed with a high-fat diet (HF) and treated with a polysaccharide extract of *Morus alba* leaves containing mannose, rhamnose, glucose, xylose, and arabinose, biochemical parameters were improved when compared to the untreated diabetic group and the control group. Among the observed results, there was an improvement in glucose tolerance, a restoration of hepatic glycogen levels, an increase in insulin levels, and an improvement of hepatic oxidative stress. In addition, there was expression of the insulin receptor substrate 2 (IRS-2), and a reduction in protein tyrosine phosphatase 1B (PTP1B) in the liver, which are two modulators with opposite functions in insulin signaling [57].

The administration of an ethanolic extract from leaves of *Morus alba* at two doses (150 and 300 mg/kg) to diabetic animals for 12 weeks resulted in hypoglycemic effects. In addition to blood glucose reduction, there was a reduction in serum lipid levels, such as total cholesterol and triglycerides. It also provided protection against oxidative damage in the kidneys [53]. A similar result was found in a study where rats received hydroethanolic extracts from the leaves of *Morus alba* for 12 weeks, and obtained a reduction of insulin, total cholesterol, and triglycerides [3].

The supply of HF diets and supplementation with two types of extracts (acetylic and ethanolic at 65%) of leaves of *Morus alba* for diabetic rats over seven weeks showed hypoglycemic effects and an increased insulin sensitivity. The ethanolic extract obtained the best results [55]. Similar to the other results, diabetic rats that received a HF diet treated with ethanolic extract at different concentrations for four weeks presented a greater reduction in fasting glycaemia levels when compared to those that receiving acetone extracts or dried leaves, these being added to the diet (22 mg/g HF diet) [56]. This can be attributed to the higher amounts of total phenols, flavonoids, chlorogenic acid, caffeic acid, rutin, and other phytochemical compounds present in ethanolic extracts [55,56].

The consumption of tea of *Morus alba* leaves is common among Asian populations, due to its effects on blood pressure control, as an anti-inflammatory, and as a renal protector. However, diabetic rats receiving different tea concentrations (0.25 and 0.50%) of leaves of *Morus alba* had no antidiabetic effects for four weeks, with a worsening of glucose tolerance. The authors attributed this result to the different concentrations used in other studies, besides the environmental influences that the different sites exert, and that directly affect the chemical compositions of the plants. On the other hand, hypolipidemic effects, such as the reduction of total cholesterol, LDL-c, and triglycerides, were observed [54]. 

Women with impaired glucose tolerance, receiving 75 g of cooked rice coated with leaf extracts of *Morus alba*, were reported to have reduced postprandial blood glucose levels when compared to the group receiving plain rice. This result was maintained for 1–2 h post-meal, indicating the hypoglycemic effects of rice coated with *Morus alba*. The authors mentioned that hypoglycemic effects may occur because of the presence of DNJ, which is present in various bioactive substances, and it found in the leaves of *Morus alba* [36]. This substance inhibits the enzymatic action of α-glucosidase and mannose dehydrogenase, which decompose disaccharides into glucose, thus affecting the digestion and absorption of carbohydrates, and suppressing postprandial hyperglycemia [56].

It is further noted that the hypoglycemic effects caused by the extracts of *Morus alba*, whether from their fruits or leaves, are positive for the treatment of subjects with impaired glucose tolerance or diabetic individuals. In addition, they interfere by improving the effects of metabolic changes during the disease, such as hypercholesterolemia and hypertriglyceridemia [36,53].

The ability of mulberry anthocyanin extract (MAE) to improve insulin resistance was evaluated in HepG2 cells and in C57BL6/J mice with genetic backgrounds (db/db). In vitro, MAE improved insulin resistance in HepG2 cells and increased glucose uptake and glycogen content. The enzymatic activities of phosphoenolpyruvate carboxykinase (PEPCK) and glucose-6-phosphatase (G6Pase) were reduced due to PPARγ coactivator 1α (PGC-1α) and forkhead box protein O1 (FOXO1) inhibition. In addition, phosphorylation of protein kinase B (AKT) and glycogen synthase kinase-3β (GSK3β) in model cells was recovered after treatment with MAE, leading to a positive regulation of glycogen synthase 2 (GYS2), and this effect was blocked by phosphatidylinositol-3-kinase (PI3K). In vivo, MAE supplementation (50 and 125 mg/kg body weight per day) markedly decreased blood glucose, serum insulin, leptin, triglyceride, and cholesterol levels and increased levels of adiponectin in db/db mice. Improvement of metabolic parameters was in part associated with the impact of MAE on activating AKT and downstream targets in liver, skeletal muscle, and adipose tissues [59].

## 6. Therapeutic Use of the Species *Morus* for Inflammation

Pharmacological studies have shown that mulberry may provide health benefits through immunomodulatory and anti-inflammatory effects [60], as well as anti-nociceptive effects [30]. Inflammation is an immune defense mechanism, and in this process, a variety of chemical mediators, which are also underlying pain, are released, including cytokines [31].

The anti-inflammatory activity of the methylene chloride extract of leaves of *Morus nigra*, investigated at different doses (100 and 300 mg/kg) in adult male rats, was evidenced by the reduction of carrageenan-induced edema in the legs of animals, and by a significant inhibition of granulomatous tissue formation. The chemical compounds isolated from the extract of *Morus nigra*, betulinic acid, β-sitosterol, and germanicol may have been responsible for the positive actions obtained [61].

The anti-nociceptive activities of fruit extracts of *Morus nigra*, *Morus mongolica*, and *Morus alba* were found in mice of the Kunming lineage. The cyanoidin-3-*O*-glycoside (C3G) anthocyanin and the flavonoids rutin (Ru) and isoquercetin (IQ), present especially in *Morus nigra*, were the main constituents active in the anti-nociceptive process. They contributed to the significant reduction of inflammatory cytokine IL-6 levels, the inhibition of iNOS synthesis, and increased expression of the anti-inflammatory cytokine IL-10. These inflammatory biomarkers are associated with pain [30].

The anti-inflammatory and anti-nociceptive properties of total flavonoids found in fruits of *Morus nigra* were also analyzed, based on ear edema and paw edema in mice induced by xylene and carrageenan, respectively, and formalin test and enzyme-linked immunosorbent assay (ELISA) to detect pro-inflammatory cytokines (IL-1β, TNF-α, IFN-γ, and NO) in the serum of mice. The results evidenced that concentrations of 50 and 100 mg of total flavonoids of *Morus nigra* per kilogram of animal weight have anti-inflammatory and analgesic effects, which may correlate with their antioxidant activity and inhibit or significantly eliminate pro-inflammatory cytokines [31].

In the inflammatory process, a variety of chemical mediators are released from the damaged tissue, including excitatory amino acids, hydrogen ions, peptides, lipids, and cytokines [62]. Pro-inflammatory cytokines damage tissues, causing redness, heat, swelling, and pain, which are the classic clinical signs of inflammation [63].

Inflammation in obese and diabetic individuals has been frequently reported, and traditionally, different parts of *Morus alba* are used for the treatment of these symptoms [35].

The leaf hydroethanolic extract of *Morus alba*, when administered for 12 weeks in animals receiving a HF diet at a dose of 200 mg/kg of body weight, was responsible for reducing serum lipids and inflammatory cytokines, which are commonly high in obese individuals. Among the cytokines that showed a reduction were IL-1β, IL-4, and TNF-α. A reduction in ALT and AST levels was also observed. They are important serum markers of liver function [3]. The reduction in ALT and AST levels was also satisfactory in diabetic rats receiving the leaf extract of *Morus alba* (480 mg/kg) for 21 days [36].

The effects of different doses of ethanolic extract of leaves of *Morus alba* under acute inflammation were evaluated, and they showed a reduction in leukocyte migration when compared to the negative control (saline solution). In addition, the results were shown to be similar to the anti-inflammatory indomethacin. These results were attributed to the presence of chlorogenic acid and flavonoids that control the expression of NF^k^β, and that prevent oxidative stress related to inflammation. They also play an important role in the signaling of inflammatory cytokines [35].

The effects of inflammation of leaves and fruit extracts of *Morus alba* were also observed in obese animals after 12 weeks of treatment, where there was a normalization of the NLRP3 inflammatory protein during the early stages of healing and accelerated healing when compared to animals that did not receive supplementation with the extracts. NLRP3 plays a key role in the induction of inflammation progression, since it triggers inflammatory cascades and decreases angiogenesis during the wound healing stages. Its control generates an improvement in the installed inflammation [64].

Peng et al. [65] also found effects under the inflammatory markers during the administration of different concentrations (0.5, 1, and 2%) of leaf extracts of *Morus alba* in animals with non-alcoholic liver steatosis. As a result, supplementation was found to reduce the inflammatory marker TNF-α, but it did not change the IL-6 levels. There was also a reduction in the serum leptin levels, and an increase in adiponectin levels. This increase in adiponectin, which acts as an anti-inflammatory agent, has been considered as a factor for the improvement of inflammation caused by steatosis in animals [65].

These results suggest that extracts from different parts of the species *Morus alba* and *nigra* show a reduction in the markers of inflammation in different parts of the organism.

## 7. Conclusions

The species *Morus* (mulberry) presents a nutraceutical potential. It is therefore a promising alternative for medicinal products based on medicinal plants. The studies presented in this review evidenced that, due to phenolic, flavonoid, and anthocyanin compounds, the species *Morus* provides health benefits related, for example, to immunomodulatory, anti-inflammatory, and anti-nociceptive activity. It may also serve as a source for the development of new therapeutic proposals for the prevention or treatment of metabolic dysfunctions related to obesity, especially DM2.

## Figures and Tables

**Table 1:** Description of the nutrients of the fruits of *Morus nigra*, *alba*, and *rubra*.

Chemical Composition	*Morus alba*	*Morus nigra*	*Morus rubra*	Reference
Lipid (%)	1.10	0.95	0.85	[16]
Linoleic acid (%)	57.26	61.85	43.39	[16]
Palmitic acid (%)	22.42	12.06	24.79	[16]
Oleic acid (%)	10.49	14.75	-	[16]
Protein (%)	10.15–13.33	8.9–10.85	-	[20]
P (mg/100 g)	247	232	226	[16]
K (mg/100 g)	1668	922	834	[16]
Ca (mg/100 g)	152	132	132	[16]
Mg (mg/100 g)	106	106	115	[16]
Fe (mg/100 g)	4.2	4.2	4.5	[16]
Na (mg/100 g)	60	59	61	[16]
Mn (mg/100 g)	3.8	4.2	4.0	[16]
Zn (mg/100 g)	2.8	3.2	3.2	[16]
Cu (mg/100 g)	0.5	0.4	0.4	[16]
Se (mg/1000 g)	0.005	0.008	-	[18]
Vitamin C (mg/100 mL)	22.4	21.8	19.4	[16]

Note: “-” compound not measured in the *Morus rubra* species.

**Table 2:** Description of the phytochemicals of the species *Morus nigra*, *alba*, and *rubra*.

Phytochemicals	*Morus alba*	*Morus nigra*	*Morus rubra*	Reference
Total phenolics (mg GAE/100 g)	181	1422	1035	[16]
Total flavonoids (mg GAE/100 g)	29	276	219	[16]
Cyanidin-3-*O*-glucoside (mg/g)	ND	8.2168	-	[30]
Cyanidin-3-*O*-rutinoside (mg/g)	ND	2.8578	-	[30]
Pelargonidin-3-*O*-glucoside (mg/g)	ND	0.2539	-	[30]
Quercetin-3-*O*-rutinlside (mg/g)	0.0816	0.4498	-	[30]
Isoquercetin (mg/g)	0.0631	0.1639	-	[30]
Morin hydrate (mg/g)	<0.0001	0.0002	-	[30]
Quercetin (mg/g)	0.0036	0.0716	-	[30]
Kaempferol (mg/g)	ND	<0.0001	-	[30]
Total benzoic acid derivatives (mg/g dry wt)	2.33–0.81	2.55–0.48	-	[20]
Total cinnamic acid derivatives (mg/g dry wt)	1.29–0.25	3.74–0.60	-	[20]
Total anthocyanins (mg/g dry wt)	-	1.88–0.01	-	[20]

Note: “-” compound not measured in the *Morus rubra* species.

**Table 3:** Effects of administration of the plant *Morus alba* L. on diabetes mellitus.

Host	Treatment	Effects	Reference
Wistar Rats	Four groups for seven weeks: Diabetes control group Metformin group Group extract polysaccharides of fruits, 50% Group extract polysaccharides of fruits, 90%	- Reduction of fasting glycaemia and glycated serum protein - Reduction of total cholesterol and triglycerides	[25]
C57BL/6 mice	Five groups for 12 weeks: Control group Group High Fat (HF) Group HF leaf extract, *M. alba* Group HF beta-glucan Group HF extract *M. alba* + beta-glucan	- Insulin reduction - Reduction of total cholesterol and triglycerides - Reduction of inflammatory markers	[3]
STZ-mice	Mouse group (diabetic control, DC group, 10 mice) and mulberry leaf powder (*M. alba*) gavage group (DD group, 10 mice)	-Activation of target proteins, like UBD, IGF2, Grb10, and IRS - Reduction of fasting blood glucose and insulin levels	[52]
Glucose tolerance humans	Four groups in two meals: Control group A + rice coated with extract of *M. alba* leaves Glucose-tolerant group + rice coated with extract of *M. alba* leaves Control group B + plain rice group with glucose tolerance + plain rice	- Reduced postprandial blood glucose	[36]
Male Sprague-Dawley rats	Three groups for 21 days: Control group Group leaf extract *M. alba* Acarbose group	- Reduced blood glucose - Reduced levels of enzymes alanine transaminase and aspartate transaminase	[36]
Male Wistar rats	Five groups for 12 weeks: Control group Diabetes control group Metformin group Group leaf extract (150 mg/kg) Group leaf extract (300 mg/kg)	- Reduced blood glucose - Reduced triglycerides, LDL-c, VLDL-c and total cholesterol	[53]
Male Sprague-Dawley rats	Four groups for four weeks: Control group Diabetes control group Group dose 0.25% Group dose 0.5%	- No hypoglycemic effects - Reduction of total cholesterol, LDL-c and triglycerides	[54]
Male C57BL/KsJ-db/db mice	Three groups for six weeks: Diabetes control group Group rosiglitazone Group methanolic extract of fruits of *M. alba*	- Reduced fasting blood glucose - Increased insulin sensitivity and AMPK and GLUT4 levels	[14]
Male Wistar rats	Five groups for four weeks: Control group Control group HF Group HF diabetes Group HF diabetes leaf acetone extract Group HF diabetes ethanolic leaf extract	- Reduced fasting blood glucose - Increased insulin sensitivity	[55]
Male Wistar rats	Six groups for four weeks: Control group Control group HF Group HF diabetes Group HF diabetes acetone extract (6 mg/g HF diet) Group HF diabetes ethanolic extract (6 mg/g HF diet) Group HF diabetes dry leaves (22 mg/g HF diet)	- Reduced fasting blood glucose - Increased insulin sensitivity - Improvement of oxidative state	[56]
Male Wistar Rats	Three groups for six weeks: Control group Diabetes control HF group Group HF diabetes polysaccharide extract MLPII	- Improved glucose tolerance - Restoration of hepatic glycogen and insulin - Improvement in oxidative stress	[57]

Note: HF: High Fat; Polysaccharide MLPII: water-soluble polysaccharide extracted from mulberry leaves.

## References

[1] Hosseinzadeh S., Jafarikukhdan A., Hosseini A., Armand R. (2015). The application of Medicinal Plants in Tradicional and Modern Medicine: A Review of Thymus vulgaris. Int. J. Clin. Med..

[2] Thaipitakwong T., Numhom S., Aramwit P. (2018). Mulberry leaves and their potential effects against cardiometabolic risks: A review of chemical compositions, biological properties and clinical efficacy. Pharm. Biol..

[3] Xu J., Wang X., Cao K., Dong Z., Feng Z., Liu J. (2017). Combination of β-glucan and *Morus alba* L. Leaf Extract Promotes Metabolic Benefits in Mice Fed a High-Fat Diet. Nutrients.

[4] Kumar V., Chauhan S. (2008). Mulberry: Life enhancer. J. Med. Plant. Res..

[5] Mena P., Sánchez-Salcedo E.M., Tassoti M., Martínez J.J., Hernández F., Del Rio D. (2016). Phytochemical evaluation of eight white (*Morus alba* L.) and black (*Morus nigra* L.) mulberry clones grown in Spain based on UHPLC-ESI-MSn metabolomic profiles. Food Res. Int..

[6] Sánchez-Salcedo E.M., Amorós A., Hernández F., Martínez J.J. (2017). Physicochemical Properties of White (*Morus alba*) and Black (*Morus nigra*) Mulberry Leaves, a New Food Supplement. J. Food Nutr. Res..

[7] Fresno M., Alvarez R., Cuesta N. (2011). Toll-like receptors, inflammation, metabolism and obesity. Arch. Physiol. Biochem..

[8] Asghar A., Sheikh N. (2017). Role of immune cells in obesity induced low grade inflammation and insulin resistance. Cell. Immunol..

[9] Petersen K.F., Shulman G.I. (2006). Etiology of Insulin Resistance. Am. J. Med..

[10] Mohamed S. (2014). Functional foods against metabolic syndrome (obesity, diabetes, hypertension and dyslipidemia) and cardiovasular disease. Trends Food Sci. Technol..

[11] Konno K., Ono H., Nakamura M., Tateishi K., Hirayama C., Tamura Y., Hattori M., Koyama A., Kohno K. (2006). Mulberry latex rich in antidiabetic sugar-mimic alkaloids forces dieting on caterpillars. Proc. Natl. Acad. Sci. USA.

[12] Asano N. (2009). Sugar-mimicking glycosidase inhibitors: Bioactivity and application. Cell. Mol. Life Sci..

[13] Liu H.Y., Wang J., Ma J., Zhang Y.Q. (2016). Interference effect of oral administration of mulberry branch bark powder on the incidence of type II diabetes in mice induced by streptozotocin. Food Nutr. Res..

[14] Choi K.H., Lee H.A., Park M.H., Han J.S. (2016). Mulberry (*Morus alba* L.) Fruit Extract Containing Anthocyanins Improves Glycemic Control and Insulin Sensitivity via Activation of AMP-Activated Protein Kinase in Diabetic C57BL/Ksj-db/db Mice. J. Med. Food..

[15] Ahn E., Lee J., Jeon Y.H., Choi S.W., Kim E. (2017). Anti-diabetic effects of mulberry (*Morus alba* L.) branches and oxyresveratrol in streptozotocin-induced diabetic mice. Food Sci. Biotechnol..

[16] Ercisli S., Orhan E. (2007). Chemical composition of white (*Morus alba*), red (*Morus rubra*) and black (*Morus nigra*) mulberry fruits. Food Chem..

[17] Imran M., Khan H., Shah M., Khan R., Khan F. (2010). Chemical composition and antioxidant activity of certain Morus species. J. Zhejiang Univ. Sci. B..

[18] Jiang Y., Nie W.J. (2015). Chemical properties in fruits of mulberry species from the Xinjiang province of China. Food Chem..

[19] Iqbal S., Younas U., Sirajuddin, Chan K.W., Sarfraz R.A., Uddin K. (2012). Proximate Composition and Antioxidant Potential of Leaves from Three Varieties of Mulberry (Morus sp.): A Comparative Study. Int. J. Mol. Sci..

[20] Sánchez-Salcedo E.M., Mena P., García-Viguera C., Martínez J.J., Hernández F. (2015). Phytochemical evaluation of white (*Morus alba* L.) and black (*Morus nigra* L.) mulberry fruits, a starting point for the assessment of their beneficial properties. J. Funct. Foods.

[21] Chen F., Nakashima N., Kimura I., Kimura M. (1995). Hypoglicemic activity and mechanisms of extracts from mulberry leaves (folium mori) and cortex mori radicis in streptozotocin-induced diabetic mice. Yakugaku Zasshi.

[22] Souza G.R., Oliveira-Junior R.G., Diniz T.C., Branco A., Lima-Saraiva S.R.G., Guimarães A.L., Oliveira A.P., Pacheco A.G.M., Silva M.G., Moraes-Filho M.O. (2017). Assessment of the antibacterial, cytotoxic and antioxidant activities of *Morus nigra L*. (Moraceae). Braz. J. Biol..

[23] Ibarreta D., Daxenberger A., Meyer H.H. (2001). Possible health impact of phytoestrogens and xenoestrogens in food. APMIS.

[24] Jefferson A. (2003). Dietary phytoestrogens—A role in women’s health. Nutr. Food Sci..

[25] Jiao Y., Wang X., Jiang X., Kong F., Wang S., Yan C. (2017). Antidiabetic effects of *Morus alba* fruit polysaccharides on high-fat dietand streptozotocin-induced type 2 diabetes in rats. J. Ethnopharmacol..

[26] Natic M.M., Dabic D.C., Papetti A., Aksic M.M.F., Ognjanov V., Ljubojevic M., Tesic Z. (2015). Analysis and characterisation of phytochemicals in mulberry (Morus alba L.) fruits grown in Vojvodina, North Serbia. Food Chem..

[27] Sharma S.B., Tanwar R.S., Rini A.C., Singh U.R., Gupta S., Shukla S.K. (2010). Protective effect of *Morus rubra* L. leaf extract on diet-induced atherosclerosis in diabetic rats. Indian J. Biochem. Biophys..

[28] Bown D. (1995). Encyclopedia of Herbs and Their Uses.

[29] Sharma S.B., Gupta S., Ac R., Singh U.R., Rajpoot R., Shukla S.K. (2010). Antidiabetogenic action of *Morus rubra* L. leaf extract in streptozotocin-induced diabetic rats. J. Pharm. Pharmacol..

[30] Chen H., Yu W., Chen G., Meng S., Xiang Z., He N. (2017). Antinociceptive and antibacterial properties of anthocyanins and flavonols from fruits of black and non-black Mulberries. Molecules.

[31] Chen H., Pu J., Liu D., Yu W., Shao Y., Yang G., Xiang Z., He N. (2016). Anti-Inflammatory and antinociceptive properties of flavonoids from the fruits of black mulberry (*Morus nigra* L.). PLoS ONE.

[32] Zhang Y., Du W., Zhang X., Zhao H., Wang Y. (2017). Antioxidant activity and the potential for cholesterol-lowering of phenolic extract of Morus alba, Morus multicaulis, and Morus laevigata leaves from Yunnan (China). J. Food Biochem..

[33] Arfan M., Khan R., Rybarczyk A., Amarowicz R. (2012). Antioxidant Activity of Mulberry Fruit Extracts. Int. J. Mol. Sci..

[34] Radojković M.M., Zeković Z.P., Vidović S.S., Kočar D.D., Mašković P.Z. (2012). Free radical scavenging activityandtotal phenolic and flavonoid contents of mulberry (*Morus* spp. L., Moraceae) extracts. Hem. Ind..

[35] Oliveira A.M., Nascimento M.F., Ferreira M.R., Moura D.F., Souza T.G., Silva G.C., Ramos E.H., Paiva P.M., Medeiros P.L., Silva T.G. (2016). Evaluation of acute toxicity, genotoxicity and inhibitory effect on acute inflammation of an ethanol extract of *Morus alba* L. (Moraceae) in mice. J. Ethnopharmacol..

[36] Hwang S.H., Li H.M., Lim S.S., Wang Z., Hong J.S., Huang B. (2016). Evaluation of a Standardized Extract from *Morus alba* against α-Glucosidase Inhibitory Effect and Postprandial Antihyperglycemic in Patients with Impaired Glucose Tolerance: A Randomized Double-Blind Clinical Trial. Evid. Based Complement. Altern. Med..

[37] Oliveira A.M., Mesquita M.S., Silva G.C., Lima E.O., Medeiros P.L., Paiva P.M.G., Souza I.A., Napoleão T.H. (2016). Evaluation of Toxicity and Antimicrobial Activity of an Ethanolic Extract from Leaves of *Morus alba* L. (Moraceae). Evid. Based Complement. Altern. Med..

[38] Nomura T. (1988). Phenolic compounds of the mulberry tree and related plants. Prog. Chem. Org. Nat. Prod..

[39] Akhlaq A., Mehmood M.H., Rehman A., Ashraf Z., Syed S., Bawany S.A., Gilani A.H., Ilyas M., Siddiqui B.S. (2016). The Prokinetic, Laxative, and Antidiarrheal Effects of *Morus nigra*: Possible Muscarinic, Ca2+ Channel Blocking, and Antimuscarinic Mechanisms. Phytother. Res..

[40] Turan I., Demir S., Kilinc K., Burnaz N.A., Yaman S.O., Akbulut K., Mentese A., Aliyazicioglu Y., Deger O. (2017). Antiproliferative and apoptotic effect of *Morus nigra* extract on human prostate cancer cells. Saudi Pharm. J..

[41] Yimam M., Jiao P., Hong M., Brownell L., Lee Y.C., Kim H.J., Nam J.B., Kim M.R., Jia Q. (2017). A Botanical Composition from Morus alba, Ilex paraguariensis, and Rosmarinus officinalis for Body Weight Management. J. Med. Food..

[42] Jiang Y., Dai M., Nie W.J., Yang X.R., Zeng X.C. (2017). Effects of the ethanol extract of black mulberry (*Morus nigra* L.) fruit on experimental atherosclerosis in rats. J. Ethnopharmacol..

[43] Da Silva Júnior I., Barbosa H.M., Carvalho D.C.R., Barros R.A., Albuquerque F.P., da Silva D.H.A., Souza G.R., Souza N.A.C., Rolim L.A., Silva F.M.M. (2017). Brazilian *Morus nigra* attenuated hyperglycemia, dyslipidemia and prooxidant status in Alloxan-induced diabetic rats. Sci. World J..

[44] Yang X., Yang L., Zheng H. (2010). Hypolipidemic and antioxidant effects of mulberry (Morus alba L.) fruit in hyperlipidaemia rats. Food Chem. Toxicol..

[45] Yuan L., Kaplowitz N. (2013). Mechanisms of drug-induced liver injury. Clin. Liver Dis..

[46] Mallhi T.H., Qadir M.I., Khan Y.H., Ali M. (2014). Hepatoprotective activity of aqueous mathanolic extract of *Morus nigra* against paracetamol-induced hepatotoxicity in mice. Bangladesh J. Pharmacol..

[47] Song H., Lai J., Tang Q., Zheng X. (2016). Mulberry ethanol extract attenuates hepatic steatosis and insulin resistance in high-fat diet-fed mice. Nutr. Res..

[48] Tag H.M. (2015). Hepatoprotective effect of mulberry (*Morus nigra*) leaves extract against methotrexate induced hepatotoxicity in male albino rat. BMC Complement. Altern. Med..

[49] Jiang D.Q., Guo Y., Xu D.H., Huang Y.S., Yuan K., Lv Z.Q. (2013). Antioxidant and anti-fatigue effects of anthocyanins of mulberry juice purification (MJP) and mulberry marc purification (MMP) from different varieties mulberry fruit in China. Food Chem. Toxicol..

[50] Qi S.Z., Li N., Tuo Z.D., Li J.L., Xing S.S., Li B.B., Zhang L., Lee H.S., Chen J.G., Cui L. (2016). Effects of Morus root bark extract and active constituents on blood lipids in hyperlipidemia rats. J. Ethnopharmacol..

[51] American Diabetes Association (2017). Standards of medical care in diabetes-2017. J. Clin. Appl. Res. Educ..

[52] Ge Q., Zhang S., Chen L., Tang M., Liu L., Kang M., Gao L., Ma S., Yang Y., Lv P. (2018). Mulberry Leaf Regulates Differentially Expressed Genes in Diabetic Mice Liver Based on RNA-Seq Analysis. Front. Physiol..

[53] Swathi P., Manjusha K.G., Vivekanand M., Ramkishan A., Bhavani B. (2017). Effect of *Morus alba* against Hyperglycemic and Hyperlipidemic activities in streptozotocin induced Diabetic Nephropathy. Biosci. Biotechnol. Res. Asia.

[54] Wilson R.D., Islam M.D.S. (2015). Effects of white mulberry (*Morus Alba*) leaf tea investigated in a type 2 diabetes model of rats. Acta Pol. Pharm..

[55] Król E., Jeszka-Skowron M., Krejpcio Z., Flaczyk E., Wójciak R.W. (2016). The Effects of Supplementary Mulberry Leaf (*Morus alba*) Extracts on the Trace Element Status (Fe, Zn and Cu) in Relation to Diabetes Management and Antioxidant Indices in Diabetic Rats. Biol. Trace Elem. Res..

[56] Jeszka-Skowron M., Flaczyk E., Jeszka J., Krejpcio Z., Król E., Buchowski M.S. (2014). Mulberry leaf extract intake reduces hyperglycaemia in streptozotocin (STZ)-induced diabetic rats fed high-fat diet. J. Funct. Foods..

[57] Ren C., Zhang Y., Cui W., Lu G., Wang Y., Gao H., Huang L., Um Z. (2015). A polysaccharide extract of mulberry leaf ameliorates hepatic glucose metabolism and insulin signaling in rats with type 2 diabetes induced by high fat-diet and streptozotocin. Int. J. Biol. Macromol..

[58] Araújo C.M., Lúcio K.P., Silva M.E., Isoldi M.C., de Souza G.H., Brandão G.C., Schulz R., Costa D.C. (2015). *Morus nigra* leaf extract improves glycemic response and redox profile in the liver of diabetic rats. Food Funct..

[59] Yan F., Dai G., Zheng X. (2016). Mulberry anthocyanin extract ameliorates insulin resistance by regulating PI3K/AKT pathway in HepG2 cells and db/db mice. J. Nutr. Biochem..

[60] Liu C.J., Lin J.Y. (2013). Anti-inflammatory effects of phenolic extracts from strawberry and mulberry fruits on cytokine secretion profiles using mouse primary splenocytes and peritoneal macrophages. Int. Immunopharmacol..

[61] Padilha M.M., Vilela F.C., Rocha C.Q., Dias M.J., Soncini R., dos Santos M.H., Alves-da-Silva G., Giusti-Paiva A. (2010). Antiinflammatory properties of *Morus nigra* leaves. Phytother. Res..

[62] Alvarez Perez Gil A.L., Barbosa Navarro L., Patipo Vera M., Petricevich V.L. (2012). Anti-inflammatory and antinociceptive activities of the ethanolic extract of *Bougainvillea xbuttiana*. J. Ethnopharmacol..

[63] Wang Y., Chen P., Tang C., Wang Y., Li Y., Zhang H. (2014). Antinociceptive and anti-inflammatory activities of extract and two isolated flavonoids of *Carthamus tinctorius* L.. J. Ethnopharmacol..

[64] Eo H., Lim Y. (2015). Combined Mulberry Leaf and Fruit Extract Improved Early Stage of Cutaneous Wound Healing in High-Fat Diet-Induced Obese Mice. J. Med. Food..

[65] Peng C.H., Lin H.T., Chung D.J., Huang C.N., Wang C.J. (2018). Mulberry Leaf Extracts prevent obesity-induced NAFLD with regulating adipocytokines, inflammation and oxidative stress. J. Food Drug Anal..

